# Automated environmental metagenomics using Oxford nanopore sequencing

**DOI:** 10.1186/s12864-025-11989-w

**Published:** 2025-09-26

**Authors:** Harry T. Child, Lucy Wierzbicki, Gabrielle R. Joslin, Katherine Roper, Qiellor Haxhiraj, Richard K. Tennant

**Affiliations:** 1 Geography, Faculty of Environment, Science and Economy, Amory Building, Rennes Drive, Exeter, Devon, EX4 4RJ UK; 2 Agilent Technologies LDA UK Limited, 5500 Lakeside, Cheadle Royal Business Park, Stockport, Cheshire, SK8 3GR UK

**Keywords:** Automation, Long-read sequencing, Metagenomics, Oxford nanopore, Library preparation, Soil

## Abstract

**Background:**

Long-read sequencing has revolutionised metagenomics through improved metagenome assembly, taxonomic classification and functional characterisation. Automation can enhance the throughput, reproducibility, and accuracy of library preparation. However, the validation of automated library preparation protocols remains undetermined for metagenomic workflows, which are particularly sensitive to methodological perturbation. Here, we compare long-read metagenomic sequencing of environmental samples through parallel manual and automated protocols.

**Results:**

Although automated library preparation led to minor reduction in read and contig lengths, taxonomic classification rate and alpha diversity was slightly higher than manual libraries, including the detection of more rare taxa. Despite this, no significant difference in microbial community structure was identified between manual and automated libraries.

**Conclusions:**

Despite minor differences in sequencing and classification metrics, automated and manual library preparation resulted in comparable characterization of environmental community metagenomes. These findings demonstrate the suitability of automation for high-throughput long-read metagenomics, with broad applicability to automated long-read sequencing for improved efficiency and reproducibility.

**Supplementary Information:**

The online version contains supplementary material available at 10.1186/s12864-025-11989-w.

## Background

Long-read sequencing has transformed our understanding of microbiomes through improved genome assembly, functional characterisation and taxonomic classification accuracy and precision [1–3], leading to its rapid expansion in metagenomic research [4]. Third generation sequencing methods from both Oxford Nanopore Technologies (ONT) and Pacific Biosciences (PacBio) have enabled the assembly of complete bacterial genomes from environmental and host-associated microbiomes [1, 5–7], allowing confident functional classification of unculturable microorganisms [8, 9]. Furthermore, utilisation of full-length 16 S amplicon sequencing and longer whole genome metagenomic reads provides more information for higher resolution of taxonomic classification [2, 10–12]. The enhanced capacity for multiplexing samples on Oxford Nanopore Technologies (ONT) platforms, as well as increases in potential yield and reduced costs of ONT sequencing [4], has improved the potential throughput of long read metagenomics. However, multiplexed library preparation protocols involve many pipetting steps, requiring considerable hands-on time and introducing potential for human error and inter-sample variation.

Automation using liquid handling robotics therefore has the potential to enhance the throughput, reproducibility, and accuracy of sequencing library preparation [13, 14]. However, validation of liquid handling automation for ONT protocols remains limited in the literature, with studies limited to high throughput amplicon sequencing of SARS-CoV-2 [15]. Along with clinical applications, validation of sample preparation processes is particularly important in metagenomic workflows due to the sensitivity of these analyses to perturbations from methodological bias [16, 17], which can impact the interpretation of study results [18, 19].

Here, we compared long-read metagenomic sequencing of environmental samples using either manual or automated ONT library preparation. We utilised the Bravo Automated Liquid Handing Platform (Agilent Technologies, UK), which has a 96-channel pipetting head for simultaneous execution of liquid handling steps across a 96-well plate. ONT sequencing libraries were prepared in parallel manually and on the Bravo using 24 DNA samples, extracted from soils with a range of habitat and geochemical traits. Analysis of metagenomic data revealed that while there were differences in read length, classification rate and alpha diversity between manual and automated libraries, there was minimal impact on the observed microbial community composition. Considering the benefits of reduced hands-on time, reproducibility and reliability, automated library preparation using the Bravo should be considered for increasing throughput of long-read sequencing.

## Results and discussion

**Fig. 1:**
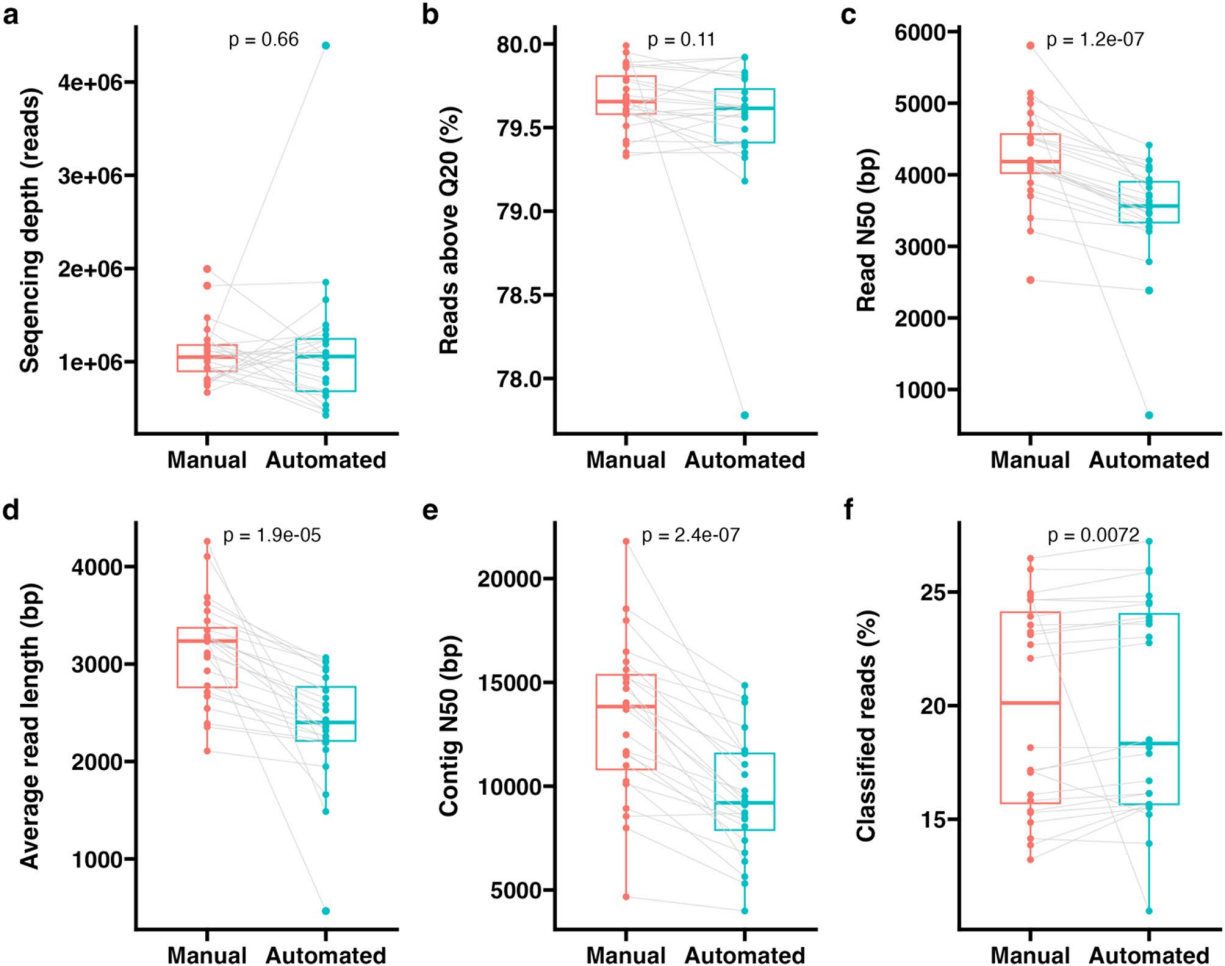
Sequencing read metrics. Boxplots comparing (**a**) sequencing read depth, **b** read length N50, **c** mean read length, **d** percentage of reads above a quality score of Q20, **e** N50 of assembled contigs and (**f**) percentage of reads classified by Kraken2 from manual or automated library preparation. Grey lines indicate paired samples prepared in parallel and results of Wilcoxon signed-rank tests are displayed

Sequencing read metrics were compared between automated and manual library preparations (Fig. 1; Supplementary Table 1). No significant difference in sequencing depth or read quality scores was identified between paired libraries (Fig. 1a-b; Supplementary Table 1). However, read length was found to be significantly longer from manually prepared libraries, with a mean difference in average length and N50 of 756 bp and 785 bp, respectively (Fig. 1c-d; Supplementary Table 1). This resulted in more contiguous assemblies from manually prepared libraries (Fig. 1e; Supplementary Table 1). However, when reads were taxonomically assigned using *Kraken2*, a small but significantly higher percentage of reads was classified from automated libraries (Fig. 1f; Supplementary Table 1), with a mean difference in classification rate of only 0.5% (excluding the outlying sample from the pasture soil).

Differences in read length and resulting assembly contiguity are likely caused by variation in bead purification steps between manual and automated protocols. While shaking to elute DNA from magnetic beads was carried out at 37 ˚ C in the manual protocol to improve elution of long fragments, as recommended in the ONT protocol, simultaneous temperature control and shaking was not possible on the Bravo. This may have caused reduced efficiency of long DNA fragment elution for the automated libraries, potentially reducing the average fragment length of automated libraries. However, the length of DNA was not analysed prior to pooling to confirm this. Meanwhile, the taxonomic classification rate may have been slightly improved in automated libraries through increased efficacy of DNA purification, leading to reduction in PCR artefacts.

Metagenome-assembled genomes (MAGs) were generated using assemblies from each sample and all medium- and high-quality MAGs were selected for further analysis. Overall, MAG recovery was poor, with 65% of libraries resulting in 0–1 medium-quality MAGs (Supplementary Table 1), likely due to insufficient sequencing depth. Although completeness and contamination across all MAGs was not significantly different between library preparation methods (Supplementary Fig. 1a-b), significantly more medium quality MAGs per sample were generated from manually prepared libraries (Supplementary Fig. 1c). This difference is mostly driven by the heath soil, which did not result in any medium quality MAGs from automated library preparation (Supplementary Table 1; Supplementary Fig. 1 d). This soil type displayed the longest read and assembly lengths of any soil type in manually prepared libraries (Supplementary Table 1), likely facilitating improved MAG generation. Meanwhile, medium quality MAG assembly was improved in automated libraries from the woodland soil (Supplementary Table 1; Supplementary Fig. 1c-d), indicating soil type-driven variability in assembly from different library preparation methods. These results indicate some improvement in MAG generation using manually prepared libraries. However, considering long-read MAG assembly from complex metagenomic samples benefits from considerably higher sequencing depth (> 20 Gbp per sample) [1, 20, 21], high-throughput metagenomic library preparation using automation is likely to be more suited to ecological analysis through taxonomic and functional classification.

**Fig. 2:**
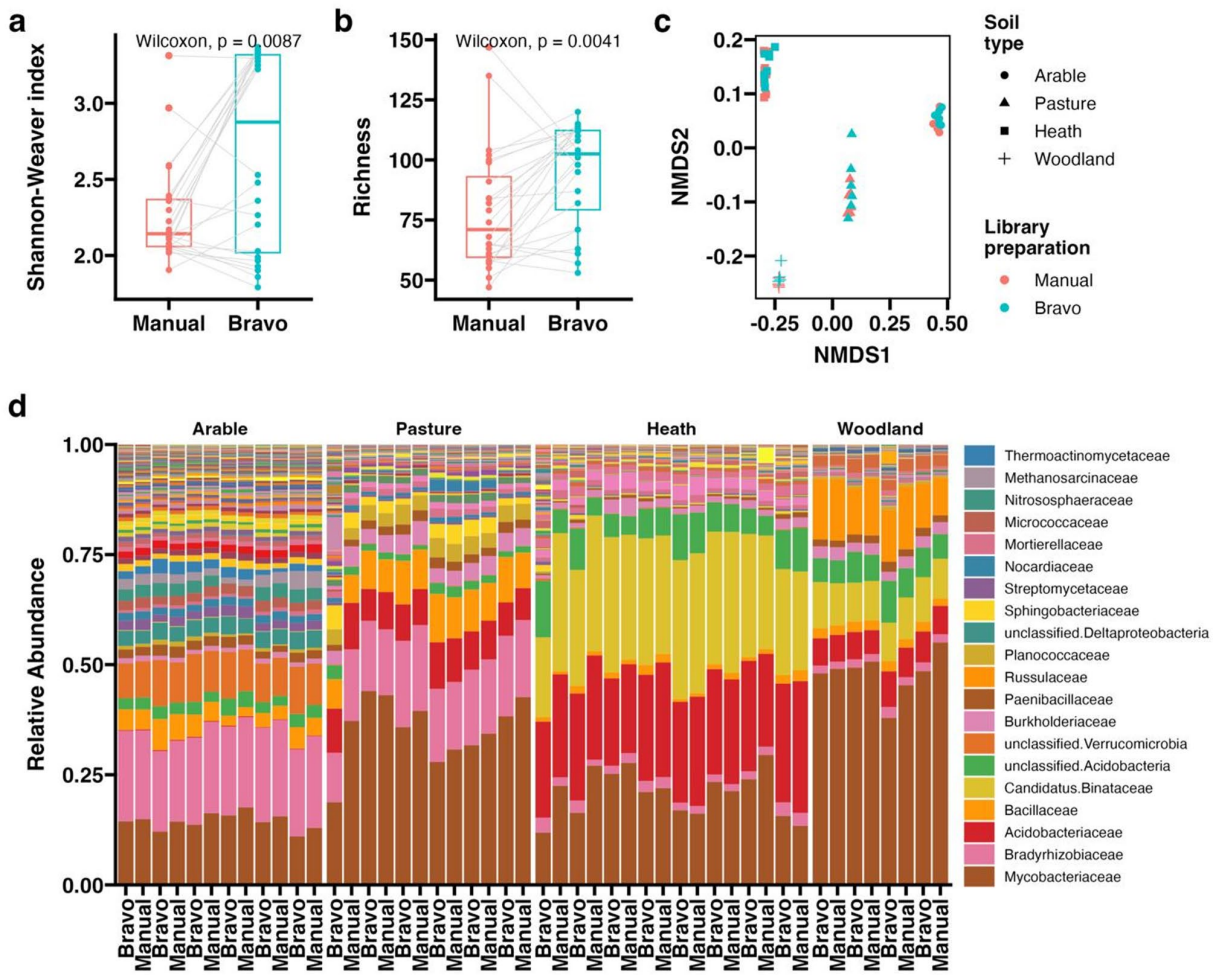
Family level microbial community analysis. Boxplots comparing alpha diversity metrics calculated at the Family taxonomic rank, including (**a**) Shannon-Weaver index and (**b**) family richness, with grey lines indicating paired samples prepared in parallel and Wilcoxon signed-rank test results displayed. **c** Non-metric Multidimensional Scaling (nMDS) plot based on Bray-Curtis distances, showing variation between the observed microbial community structure of manual and automated libraries from the four soil types. **d** Stacked bar chart showing the relative abundance of microbial families across the four soil types. Legend shows colours corresponding to the top 20 families

Ecological analyses were performed on the results of taxonomic classification at a Family level (Fig. 2). A significant increase in alpha diversity, measured as both Shannon-Weaver index and family richness, was observed in libraries prepared on the Bravo (Fig. 2a-b), which was mostly the result of the presence of rare taxa (Fig. 2d). Detection of rare microorganisms in complex samples is an important objective of many metagenomic studies, due to their importance to ecosystem functions and community dynamics [22, 23], for which the increased diversity of automated libraries observed here could provide a benefit.

Variation in microbial community structure was investigated through calculation of Bray-Curtis distances with rarefaction (Fig. 2c). Unsurprisingly, soil type was found to explain the vast majority of variation in community composition between the samples (PERMANOVA, R^2^ = 0.92, *p* < 0.001), while library preparation method or the interaction between these variables showed no significant effect (Fig. 2c). To support this, analysis within each soil type found no significant effect of library preparation method on microbial community composition at any taxonomic rank (PERMANOVA, *p* > 0.05). This indicates that minimal differences in microbial community composition were observed between manual and automated libraries, with no pattern to this variation within each soil type. Such consistency is crucial if the results from manual and automated library preparations are to be compared, considering the importance of reproducibility for interpretation of metagenomic data within and between studies.

Demonstrating reproducibility is especially important for analysis of environmental samples, such as soil, that are particularly vulnerable to perturbation by methodological variation [16, 18]. The soil matrix exhibits high spatial heterogeneity of microorganism distribution [16], as well as containing an abundance of inhibitors posing a challenge to molecular genetic analysis. Furthermore, microbial ecologists wish to characterise soil communities from a field to a continental scale [24, 25], while most soil nucleic acid extraction methods require comparably minuscule input quantities (250 µg–2 g). Considering these factors, and the statistical analysis required for deciphering differential abundance, sufficient sampling sizes and replication are crucial to uncover patterns in microbial community composition and function between sites and experimental treatments [16, 26, 27]. Automation has the potential to address these challenges of increased throughput and maintain reproducibility.

## Conclusion

Despite the identification of minor differences in sequencing metrics and detection of rare taxa between manual and automated protocols, automated library preparation had minimal impact on the microbial community characterised from parallel metagenomic analysis of soil DNA samples. Considering the benefits of reduced hands-on time, reproducibility and reliability, automated library preparation should be considered suitable for improving throughput of ONT long-read sequencing.

## Methods

Soil samples from four habitats were collected and characterised as previously described [28]. DNA was extracted using the DNeasy^®^ PowerSoil^®^ Pro Kit (Qiagen, UK), with 4–8 extractions from each soil type. DNA input into library preparations was normalised to 1 µg. Libraries were prepared using the Ligation Sequencing Kit (SQK-LSK114; ONT, UK) and PCR Barcoding Expansion 96 (EXP-PBC096; ONT, UK), with parallel preparations carried out manually, following manufacturer’s protocol (Additional file 1), and automated on the Bravo (detailed in Additional file 2) on the same samples. Between 15 and 45 ng DNA was input into PCR barcoding reactions. Parallel preparations concluded with normalised pools of barcoded libraries, which were subsequently pooled together and sequenced on the same R10.4.1 PromethION flowcell.

Sequencing yielded 125.74 Gb of data in 42.79 million reads with an N50 of 3.99 kb. Reads were basecalled and demultiplexed using *guppy* v7.1.4 and adapters were trimmed using *dorado* v0.6.0. Metagenome assembly was carried out using *metaFlye* [29], with reads input using the --nano-hq parameter. Contigs above 1 kb were binned with SemiBin2 [30], using the single_easy_bin function (with the parameters --sequencing-type = long_read --self-supervised). The resulting MAGs were assessed for quality using CheckM2 [31] and the filtered medium and high quality MAGs (genome completeness > 50% and contamination < 10%) were dereplicated to identify overlap between samples using dRep [32]. Taxonomic classification was carried out using *Kraken2* v 2.1.2 [33] against the NCBI nr database (downloaded on 09/03/24), using confidence score threshold of 0.05 to reduce the occurrence of false positives. Count tables were compiled using *MEGAN* Ultimate Edition v6.25.6 [34] and filtered to remove taxa occurring at an abundance of < 0.1% across all samples. Ecological statistics were calculated using the *vegan* v2.6-4 R package [35]. Bray-Curtis distances were calculated using the *avgdist* function with subsampling to the minimum classified read counts across samples.

## Supplementary Information

Additional file 1. Protocol checklist for ligation sequencing V14 with PCR barcoding (SQK-LSK114 with EXP-PBC001 or EXP-PBC096) on PromethION.

Additional file 2. ONT General Ligation Agilent Bravo Option B Automated User Guide

Additional file 3. Supplementary Figure 1. Analysis of medium-quality metagenome-assembled genomes. Supplementary Table 1. Table of read and assembly statistics for each sequenced sample.

## Data Availability

The datasets generated and analysed during the current study are available in the NCBI Sequence Read Archive repository, [BioProject accession PRJNA1112790](https:/www.ncbi.nlm.nih.gov/bioproject/PRJNA1112790).
