# Supplementary material for: Automated environmental metagenomics using Oxford nanopore sequencing
Source: BMC Genomics. 2025 Sep 26;26:835. doi: 10.1186/s12864-025-11989-w (PMC12465296; doi:10.1186/s12864-025-11989-w)
Supplement: Supplementary file 1 — Additional file 1. Protocol checklist for ligation sequencing V14 with PCR barcoding (SQK-LSK114 with EXP-PBC001 or EXP-PBC096) on PromethION. [file 12864_2025_11989_MOESM1_ESM.pdf]

End Prep

1. Add 45µl of DNA to an Eppendorf Twin.tec full skirt plate
2. Set up End Prep mastermix as shown in Table 1

**Table 1: End Prep Mastermix**		Volume per Number of Columns of Samples (µl)					
Component	Volume for 1 reaction (µl)	1	2	3	4	6	12
End prep buffer	7	71.1	129.9	194.9	253.1	378.0	745.9
End prep enzyme	3	30.5	55.7	83.5	108.5	162.0	319.7
Water	5	50.8	92.8	139.2	180.8	270.0	532.8
Total Volume	15	152.4	278.4	417.6	542.4	810.0	1598.4

3. Add the correct volume of End Prep Mastermix to a 1ml deep well plate as shown in Table 2

**Table 2: End Prep Mastermix Distribution**			Sample Columns					
	MM Plate Column	per Rxn	1	2	3	4	6	12
End Prep Mastermix	1	15µl	18µl	33µl	50µl	66µl	99µl	198µl

4. Add 60µl of Ampure beads to a well of a 1ml deep well plate per sample being used. For example if using 2 columns of samples, beads are added to columns 1 & 2 of the plate
5. Add 50ml of 80% ethanol to a reservoir
6. Add 50ml of nuclease-free water to a reservoir
7. Select "1_End Repair and Cleanup.rst" and set PCR plate labware and number of columns of samples
8. Select "Display Initial Workstation Setting" and add labware as indicated on form
9. Select "Run Selected Protocol"

Ligation

1. Add the correct volume of End Prep Mastermix to a 1ml deep well plate as shown in Table

**Table 3: Ligation Mastermix Distribution**			Sample Columns					
	MM Plate Column	per Rxn	1	2	3	4	6	12
Barcode Adapter	1	10µl	18µl	33µl	50µl	66µl	99µl	198µl
Blunt/TA Ligase Master Mix	2	25µl	28µl	55µl	82.5µl	110µl	165µl	330µl

2. Add 22.5µl of Ampure beads to a well of a 1ml deep well plate per sample being used. For example if using 2 columns of samples, beads are added to columns 1 & 2 of the plate
3. Add 50ml of 80% ethanol to a reservoir
4. Add 50ml of nuclease-free water to a reservoir
5. Select "2_Barcode Adapter Ligation and Cleanup.rst" and set PCR plate labware and number of columns of samples
6. Select "Display Initial Workstation Setting" and add labware as indicated on form
7. Select "Run Selected Protocol"

The “Sample Normalisation.pro” protocol can be used to normalize samples before the Adapter Barcoding step

Adapter Barcoding

1. For 1 column of samples make 100-200fmol of DNA up to 48µl with Nuclease-free water in a PCR plate. For 2 columns or more, make 100-200 fmol of DNA up to 23µl with Nuclease-free water
2. Transfer 4µl of indexes to corresponding wells of an Eppendorf Twin.tec plate per sample being used. If using 2 or more columns of samples dilute the indexes 1:2 in nuclease-free water first
3. Add the correct volume of Barcoding Mastermix to a 1ml deep well plate as shown in Table 4

Table 4: Barcoding Mastermix Distribution

			Sample Columns					
	MM Plate Column	per Rxn	1	2	3	4	6	12
LongAmp Taq 2X master mix	1	25/50µl	55µl	55µl	82.5µl	110µl	165µl	330µl

4. Select "03 Barcoding PCR v.B1.0.2.pro" and set PCR plate labware and number of columns of samples
5. Select "Display Initial Workstation Setting" and add labware as indicated on form
6. Select "Run Selected Protocol"

Barcoding Cleanup

1. For 1 column of samples add 40µl of Ampure beads to a well of a 1ml deep well plate per sample being used. For 2 or more columns of samples use 20µl instead. For example if using 2 columns of samples, beads are added to columns 1 & 2 of the plate
2. Add 50ml of 80% ethanol to a reservoir
3. Add 50ml of nuclease-free water to a reservoir
4. Select "04 AMPureXP_barcoding_pcr.B1.0.2.pro" and set PCR plate labware and number of columns of samples
5. Select "Display Initial Workstation Setting" and add labware as indicated on form
6. Select "Run Selected Protocol"

The “Sample Normalisation.pro” protocol can be used to pool libraries
