# Supplementary material for: Automated environmental metagenomics using Oxford nanopore sequencing
Source: BMC Genomics. 2025 Sep 26;26:835. doi: 10.1186/s12864-025-11989-w (PMC12465296; doi:10.1186/s12864-025-11989-w)
Supplement: Supplementary file 2 — Additional file 2. ONT General Ligation Agilent Bravo Option B Automated User Guide [file 12864_2025_11989_MOESM2_ESM.pdf]

Flow Cell Number: .... DNA Samples: ....

Before start checklist

Materials

- [ ] <100–200 fmol of each DNA sample to be barcoded in 45 µl
- [ ] OR <100–200 fmol first-round PCR product (with tailed primers) per sample
- [ ] PCR Barcoding Expansion 1-12 (EXP-PBC001)
- [ ] PCR Barcoding Expansion 1-96 (EXP-PBC096)
- [ ] Ligation Sequencing Kit V14 (SQK-LSK114)

Consumables

- [ ] Agencourt AMPure XP beads (Beckman Coulter™ cat # A63881)
- [ ] NEBNext Ultra II End repair/dA-tailing Module (NEB, E7546)
- [ ] NEBNext Quick Ligation Module (NEB, E6056)
- [ ] NEB Blunt/TA Ligase Master Mix (NEB, M0367)
- [ ] LongAmp Taq 2X Master Mix (e.g. NEB, cat # M0287)
- [ ] 1.5 ml Eppendorf DNA LoBind tubes
- [ ] 0.2 ml thin-walled PCR tubes or 0.2 ml 96-well PCR plate
- [ ] Freshly prepared 80% ethanol in nuclease-free water
- [ ] Nuclease-free water (e.g. ThermoFisher, AM9937)
- [ ] Qubit™ Assay Tubes (Invitrogen, Q32856)
- [ ] Qubit dsDNA HS Assay Kit (Invitrogen, Q32851)

Equipment

- [ ] Hula mixer (gentle rotator mixer)
- [ ] Microfuge
- [ ] Vortex mixer
- [ ] Thermal cycler
- [ ] Ice bucket with ice
- [ ] Magnetic rack
- [ ] Timer
- [ ] Qubit fluorometer (or equivalent for QC check)
- [ ] Pipettes and pipette tips Multichannel, P2, P10, P20, P100, P200, P1000

INSTRUCTIONS	NOTES/OBSERVATIONS
**End-prep**	
Prepare the NEBNext Ultra II End Repair / dA-tailing Module reagents in accordance with manufacturer's instructions, and place on ice:<br>☐ Thaw all reagents on ice.<br>☐ Flick and/or invert the reagent tubes to ensure they are well mixed.<br>Note: Do not vortex the Ultra II End Prep Enzyme Mix.<br>☐ Always spin down tubes before opening for the first time each day.<br>☐ The Ultra II End Prep Buffer may have a little precipitate. Allow the mixture to come to RT and pipette the buffer up and down several times to break up the precipitate, followed by vortexing the tube for 30 seconds to solubilise any precipitate.<br><br>Prepare the DNA in Nuclease-free water.<br>☐ Transfer <100-200 fmol DNA of each sample into a fresh 0.2 ml PCR tube or plate<br>☐ Adjust the volume to 45 µl with Nuclease-free water<br>☐ Mix thoroughly by flicking the tube to avoid unwanted shearing<br>☐ Spin down briefly in a microfuge	

Flow Cell Number: .... DNA Samples: ....

INSTRUCTIONS	NOTES/OBSERVATIONS
Set up the end-repair reaction as follows for each library:<br>☐ 45 µl <100-200 fmol DNA<br>☐ 7 µl Ultra II End-prep reaction buffer<br>☐ 3 µl Ultra II End-prep enzyme mix<br>☐ 5 µl Nuclease-free water<br><br>☐ Mix by pipetting and briefly spin down.<br><br>☐ Using a thermal cycler, incubate for 5 minutes at 20 °C and 5 minutes at 65 °C.<br><br>☐ Resuspend the AMPure XP beads by vortexing.<br><br>☐ Add 60 µl of resuspended AMPure XP beads to the end-prep reaction and mix by pipetting.<br><br>☐ Incubate at RT for 5 minutes.<br><br>☐ Prepare sufficient fresh 80% ethanol in Nuclease-free water for all of your samples. Allow enough for 400 µl per sample, with some excess.<br><br>☐ Spin down the samples and pellet on a magnet until supernatant is clear and colourless. Keep the samples on the magnet, and pipette off the supernatant.<br><br>☐ Keep the samples on the magnet and wash the beads with 200 µl of freshly prepared 80% ethanol without disturbing the pellet. Remove the ethanol using a pipette and discard.<br><br>☐ Repeat the previous step.<br><br>☐ Spin down and place the samples back on the magnetic rack. Pipette off any residual ethanol. Allow the pellet to dry for ~30 seconds, but do not dry the pellet to the point of cracking.<br><br>☐ Remove the samples from the magnet and resuspend each pellet in 16 µl Nuclease-free water. Incubate for 2 minutes at RT.<br><br>☐ Pellet the beads on a magnet until the eluate is clear and colourless.<br><br>☐ Remove eluate once it is clear and colourless. Transfer each eluted sample to a new tube or plate well.<br><br>☐ Quantify 1 µl of end-prepped DNA using a Qubit fluorometer.	
Take forward the end-prepped DNA into the next step. However, at this point it is also possible to store the sample at 4°C overnight.	
**Ligation of Barcode Adapter**	
Prepare the NEB Blunt/TA Ligase Master Mix according to the manufacturer's instructions, and place on ice:<br>☐ Thaw the reagents at RT.<br>☐ Spin down the reagent tubes for 5 seconds.<br>☐ Ensure the reagents are fully mixed by performing 10 full volume pipette mixes.<br><br>☐ Spin down the Barcode Adapter (BCA), pipette mix and place on ice.	

Flow Cell Number: .... DNA Samples: ....

INSTRUCTIONS	NOTES/OBSERVATIONS
Add the reagents in the order given below, into fresh 0.2 ml PCR tubes or 96-well plate:<br>☐ 15 µl End-prepped DNA<br>☐ 10 µl Barcode Adapter<br>☐ 25 µl Blunt/TA Ligase Master Mix<br><br>☐ Mix by pipetting and briefly spin down.<br><br>☐ Incubate the samples for 10 minutes at RT.<br><br>☐ Resuspend the AMPure XP beads by vortexing.<br><br>☐ Add 20 µl of resuspended AMPure XP beads to each sample for a 0.4X clean and mix by pipetting up and down ten times.<br><br>☐ Incubate on a Hula mixer (rotator mixer) for 5 minutes at RT.<br><br>☐ Prepare sufficient fresh 80% ethanol in Nuclease-free water for all of your samples. Allow enough for 400 µl per sample, with some excess.<br><br>☐ Place on a magnetic rack, allow beads to pellet and pipette off supernatant.<br><br>☐ Keep the samples on the magnet and wash the beads with 200 µl of freshly prepared 80% ethanol without disturbing the pellet. Remove the ethanol using a pipette and discard.<br><br>☐ Repeat the previous step.<br><br>☐ Place the samples back on the magnet. Pipette off any residual 80% ethanol. Allow to dry for ~30 seconds, but do not dry the pellet to the point of cracking.<br><br>☐ Remove the samples from the magnet and resuspend pellet in 25 µl Nuclease-free water. Incubate for 2 minutes at RT.<br><br>☐ Pellet the beads on a magnet until the eluate is clear and colourless.<br><br>Remove and retain the eluate once it is clear and colourless. Transfer each eluted sample to a fresh 0.2 ml PCR tube or plate.<br>☐ Dispose of the pelleted beads.<br><br>☐ Quantify 1 µl of the adapter ligated DNA using a Qubit fluorometer.	
Take forward the adapter ligated samples into the Barcoding PCR step. However, at this point it is also possible to store the sample at 4°C overnight.	
**Barcoding PCR**	
Please note, this protocol is written for a template input of 100–200 fmol with PCR Barcodes (BC01-96) used at a final concentration of 0.2 µM. However, the input mass and the number of PCR cycles may be adjusted as appropriate depending on the requirements of the experiment.<br><br>☐ Thaw the PCR Barcodes (BC01-96) required for your number of samples at RT. Individually mix the barcodes by pipetting, spin down, and place on ice.	

Flow Cell Number: .... DNA Samples: ....

INSTRUCTIONS	NOTES/OBSERVATIONS

IMPORTANT

☐ If using amplicon samples, ensure the samples have undergone a round of PCR with tailed primers before commencing with the protocol.

Prepare the samples in Nuclease-free water:

☐ Transfer 100-200 fmol of each sample to a clear 0.2 ml PCR tube or plate

For 1–12 samples: Adjust the volume to 48 µl with Nuclease-free water
For 13–96 samples: Adjust the volume to 24 µl with Nuclease-free water

☐ For 1–12 samples: Adjust the volume to 48 µl with Nuclease-free water
☐ For 13–96 samples: Adjust the volume to 24 µl with Nuclease-free water
☐ Mix thoroughly by flicking the tube or plate to avoid unwanted shearing
☐ Spin down briefly in a microfuge

☐ Select a unique barcode for each sample to be run together on the same flow cell.

Set up a barcoding PCR reaction as follows for each library in fresh 0.2 ml PCR tubes or plate.

☐ PCR Barcode (one of BC1-BC96, at 10 µM)
- Volume per sample for using 1–12 barcodes: 2 µl
- Volume per sample for using 13 barcodes or more: 1 µl

☐ Adapter ligated DNA or amplicons with tailed primers
- Volume per sample for using 1–12 barcodes: 48 µl
- Volume per sample for using 13 barcodes or more: 24 µl

☐ LongAmp Taq 2X master mix
- Volume per sample for using 1–12 barcodes: 50 µl
- Volume per sample for using 13 barcodes or more: 25 µl

☐ Mix by pipetting and briefly spin down.

Amplify using the following cycling conditions:

☐ Initial denaturation 3 mins @ 95 °C (1 cycle)
☐ Denaturation 15 secs @ 95 °C (12-15 (b) cycles)
☐ Annealing 15 secs (a) @ 62 °C (a) (12-15 (b) cycles)
☐ Extension dependent on length of target fragment (d) @ 65 °C (c) (12-15 (b) cycles)
☐ Final extension dependent on length of target fragment (d) @ 65 °C (1 cycle)
☐ Hold @ 4 °C

☐ Resuspend the AMPure XP beads by vortexing.

Add 0.4X volume of resuspended AMPure XP Beads to each reaction and mix by flicking the tube.

☐ AMPure XP Beads
- Volume for 100 µl samples: 40 µl
- Volume for 50 µl samples: 20 µl

☐ Incubate at RT for 5 minutes.

☐ Prepare sufficient fresh 80% ethanol in Nuclease-free water for all of your samples. Allow enough for 400 µl per sample, with some excess.

Flow Cell Number: .... DNA Samples: ....

INSTRUCTIONS	NOTES/OBSERVATIONS
☐ Place on a magnetic rack, allow beads to pellet and pipette off supernatant.<br><br>☐ Keep the samples on the magnet and wash the beads with 200 µl of freshly prepared 80% ethanol without disturbing the pellets. Remove the ethanol using a pipette and discard.<br><br>☐ Repeat the previous step.<br><br>☐ Spin down and place the samples back on the magnet. Pipette off any residual ethanol. Allow to dry for ~30 seconds, but do not dry the pellets to the point of cracking.<br><br>☐ Remove the samples from the magnetic rack and resuspend each pellet in 25 µl Nuclease-free water. Incubate for 2 minutes at RT.<br><br>☐ Pellet the beads on a magnetic rack until the eluate is clear and colourless.<br><br>☐ Remove and retain 25 µl of each eluate into clean 0.2 ml PCR tubes or plate.<br><br>☐ Quantify the barcoded library using a Qubit fluorometer and pool all barcoded libraries in the desired ratios in a 1.5 ml DNA LoBind Eppendorf tube.<br><br>☐ Prepare 1 µg of pooled barcoded libraries in 49 µl Nuclease-free water.	
This pooled library is now ready to be end-repaired and adapted for sequencing. However, at this point it is also possible to store the sample at 4°C overnight.	
**End-prep**	
☐ Thaw the AMPure XP Beads (AXP) and DNA Control Sample (DCS) at RT and mix by vortexing. Keep the beads at RT and store the DNA Control Sample (DCS) on ice.<br><br>Prepare the NEBNext Ultra II End Repair / dA-tailing Module reagents in accordance with manufacturer's instructions, and place on ice:<br>☐ Thaw all reagents on ice.<br>☐ Flick and/or invert the reagent tubes to ensure they are well mixed.<br>Note: Do not vortex the Ultra II End Prep Enzyme Mix.<br>☐ Always spin down tubes before opening for the first time each day.<br>☐ The Ultra II End Prep Buffer may have a little precipitate. Allow the mixture to come to RT and pipette the buffer up and down several times to break up the precipitate, followed by vortexing the tube for 30 seconds to solubilise any precipitate.<br><br>In a 0.2 ml thin-walled PCR tube, mix the following:<br>☐ 1 µl DNA Control Sample (DCS)<br>☐ 49 µl DNA<br>☐ 7 µl Ultra II End-prep Reaction Buffer<br>☐ 3 µl Ultra II End-prep Enzyme Mix<br><br>☐ Thoroughly mix the reaction by gently pipetting and briefly spinning down.<br><br>☐ Using a thermal cycler, incubate at 20°C for 5 minutes and 65°C for 5 minutes.	

Flow Cell Number: .... DNA Samples: ....

INSTRUCTIONS	NOTES/OBSERVATIONS
☐ Resuspend the AMPure XP Beads (AXP) by vortexing.<br>☐ Transfer the DNA sample to a clean 1.5 ml Eppendorf DNA LoBind tube.<br>☐ Add 60 µl of resuspended the AMPure XP Beads (AXP) to the end-prep reaction and mix by flicking the tube.<br>☐ Incubate on a Hula mixer (rotator mixer) for 5 minutes at RT.<br>☐ Prepare 500 µl of fresh 80% ethanol in Nuclease-free water.<br>☐ Spin down the sample and pellet on a magnet until supernatant is clear and colourless. Keep the tube on the magnet, and pipette off the supernatant.<br>☐ Keep the tube on the magnet and wash the beads with 200 µl of freshly prepared 80% ethanol without disturbing the pellet. Remove the ethanol using a pipette and discard.<br>☐ Repeat the previous step.<br>☐ Spin down and place the tube back on the magnet. Pipette off any residual ethanol. Allow to dry for ~30 seconds, but do not dry the pellet to the point of cracking.<br>☐ Remove the tube from the magnetic rack and resuspend the pellet in 61 µl Nuclease-free water. Incubate for 2 minutes at RT.<br>☐ Pellet the beads on a magnet until the eluate is clear and colourless, for at least 1 minute.<br>☐ Remove and retain 61 µl of eluate into a clean 1.5 ml Eppendorf DNA LoBind tube.	
Quantify 1 µl of eluted sample using a Qubit fluorometer.	
Take forward the repaired and end-prepped DNA into the adapter ligation step. However, at this point it is also possible to store the sample at 4°C overnight.	
**Adapter ligation and clean-up**	
IMPORTANT<br>☐ Although the recommended third-party ligase is supplied with its own buffer, the ligation efficiency of the Ligation Adapter (LA) is higher when using the Ligation Buffer (LNB) supplied in the Ligation Sequencing Kit.	
☐ Spin down the Ligation Adapter (LA) and Quick T4 Ligase, and place on ice.<br>☐ Thaw Ligation Buffer (LNB) at RT, spin down and mix by pipetting. Due to viscosity, vortexing this buffer is ineffective. Place on ice immediately after thawing and mixing.<br>☐ Thaw the Elution Buffer (EB) at RT and mix by vortexing. Then spin down and place on ice.	
IMPORTANT<br>Depending on the wash buffer (LFB or SFB) used, the clean-up step after adapter ligation is designed to either enrich for DNA fragments of >3 kb, or purify all fragments equally.<br>☐ To enrich for DNA fragments of 3 kb or longer, use Long Fragment Buffer (LFB)<br>☐ To retain DNA fragments of all sizes, use Short Fragment Buffer (SFB)	

Flow Cell Number: .... DNA Samples: ....

INSTRUCTIONS	NOTES/OBSERVATIONS
☐ Thaw either Long Fragment Buffer (LFB) or Short Fragment Buffer (SFB) at RT and mix by vortexing. Then spin down and place on ice.<br><br>In a 1.5 ml Eppendorf DNA LoBind tube, mix in the following order:<br>☐ 60 µl DNA sample from the previous step<br>☐ 25 µl Ligation Buffer (LNB)<br>☐ 10 µl NEBNext Quick T4 DNA Ligase<br>☐ 5 µl Ligation Adapter (LA)<br><br>☐ Thoroughly mix the reaction by gently pipetting and briefly spinning down.<br><br>☐ Incubate the reaction for 10 minutes at RT.<br><br>☐ Resuspend the AMPure XP beads (AXP) by vortexing.<br><br>☐ Add 40 µl of resuspended AMPure XP Beads (AXP) to the reaction and mix by flicking the tube.<br><br>☐ Incubate on a Hula mixer (rotator mixer) for 5 minutes at RT.<br><br>☐ Spin down the sample and pellet on a magnet. Keep the tube on the magnet, and pipette off the supernatant when clear and colourless.<br><br>☐ Wash the beads by adding either 250 µl Long Fragment Buffer (LFB) or 250 µl Short Fragment Buffer (SFB). Flick the beads to resuspend, spin down, then return the tube to the magnetic rack and allow the beads to pellet. Remove the supernatant using a pipette and discard.<br><br>☐ Repeat the previous step.<br><br>☐ Spin down and place the tube back on the magnet. Pipette off any residual supernatant. Allow to dry for ~30 seconds, but do not dry the pellet to the point of cracking.<br><br>☐ Remove the tube from the magnetic rack and resuspend the pellet in 25 µl Elution Buffer (EB). Spin down and incubate for 10 minutes at RT. For high molecular weight DNA, incubating at 37°C can improve the recovery of long fragments.<br><br>☐ Pellet the beads on a magnet until the eluate is clear and colourless, for at least 1 minute.<br><br>☐ Remove and retain 25 µl of eluate containing the DNA library into a clean 1.5 ml Eppendorf DNA LoBind tube.	
Quantify 1 µl of eluted sample using a Qubit fluorometer.	
IMPORTANT<br>☐ We recommend loading 20 fmol of this final prepared library onto the R10.4.1 flow cell.	
☐ Prepare 20 fmol of your final library to 32 µl with Elution Buffer (EB).	
The prepared library is used for loading into the flow cell. Store the library on ice or at 4°C until ready to load.	

Flow Cell Number: .... DNA Samples: ....

INSTRUCTIONS	NOTES/OBSERVATIONS
**Priming and loading the PromethION Flow Cell**	
IMPORTANT<br>☐ This kit is only compatible with R10.4.1 flow cells (FLO-PRO114M).	
☐ Thaw the Sequencing Buffer (SB), Library Beads (LIB) or Library Solution (LIS, if using), Flow Cell Tether (FCT) and Flow Cell Flush (FCF) at RT before mixing by vortexing. Then spin down and store on ice.<br><br>To prepare the flow cell priming mix, combine Flow Cell Tether (FCT) and Flow Cell Flush (FCF), as directed below. Mix by vortexing at RT.<br>☐ 1,170 µl Flow Cell Flush (FCF)<br>☐ 30 µl Flow Cell Tether (FCT)	
IMPORTANT<br>☐ After taking flow cells out of the fridge, wait 20 minutes before inserting the flow cell into the PromethION for the flow cell to come to RT. Condensation can form on the flow cell in humid environments. Inspect the gold connector pins on the top and underside of the flow cell for condensation and wipe off with a lint-free wipe if any is observed. Ensure the heat pad (black pad) is present on the underside of the flow cell.	
For PromethION 2 Solo, load the flow cell(s) as follows:<br>☐ Place the flow cell flat on the metal plate.<br>☐ Slide the flow cell into the docking port until the gold pins or green board cannot be seen.<br><br>For the PromethION 24/48, load the flow cell(s) into the docking ports:<br>☐ Line up the flow cell with the connector horizontally and vertically before smoothly inserting into position.<br>☐ Press down firmly onto the flow cell and ensure the latch engages and clicks into place.	
IMPORTANT<br>☐ Insertion of the flow cells at the wrong angle can cause damage to the pins on the PromethION and affect your sequencing results. If you find the pins on a PromethION position are damaged, please contact support@nanoporetech.com for assistance.	
☐ Turn the valve clockwise to expose the inlet port.	
IMPORTANT<br>☐ Take care when drawing back buffer from the flow cell. Do not remove more than 20-30 µl, and make sure that the array of pores are covered by buffer at all times. Introducing air bubbles into the array can irreversibly damage pores.	
After opening the inlet port, draw back a small volume to remove any air bubbles:<br>☐ Set a P1000 pipette tip to 200 µl.<br>☐ Insert the tip into the inlet port.<br>☐ Turn the wheel until the dial shows 220-230 µl, or until you see a small volume of buffer entering the pipette tip.<br><br>☐ Load 500 µl of the priming mix into the flow cell via the inlet port, avoiding the introduction of air bubbles. Wait five minutes. During this time, prepare the library for loading using the next steps in the protocol.<br><br>☐ Thoroughly mix the contents of the Library Beads (LIB) by pipetting.	

Flow Cell Number: .... DNA Samples: ....

INSTRUCTIONS	NOTES/OBSERVATIONS
IMPORTANT<br>☐ The Library Beads (LIB) tube contains a suspension of beads. These beads settle very quickly. It is vital that they are mixed immediately before use.	
In a new 1.5 ml Eppendorf DNA LoBind tube, prepare the library for loading as follows:<br>☐ 100 µl Sequencing Buffer (SB)<br>☐ 68 µl Library Beads (LIB) thoroughly mixed before use, or Library Solution (LIS)<br>☐ 32 µl DNA library<br>☐ Complete the flow cell priming by slowly loading 500 µl of the priming mix into the inlet port.<br>☐ Mix the prepared library gently by pipetting up and down just prior to loading.<br>☐ Using a P1000, insert the pipette tip into the inlet port and load 200 µl of library.<br>☐ Close the valve to seal the inlet port.	
IMPORTANT<br>☐ Install the light shield on your flow cell as soon as library has been loaded for optimal sequencing output.	
If the light shield has been removed from the flow cell, install the light shield as follows:<br>☐ Align the inlet port cut out of the light shield with the inlet port cover on the flow cell. The leading edge of the light shield should sit above the flow cell ID.<br>☐ Firmly press the light shield around the inlet port cover. The inlet port clip will click into place underneath the inlet port cover.	
Close the PromethION lid when ready to start a sequencing run on MinKNOW.	
**Flow cell reuse and returns**	
☐ After your sequencing experiment is complete, if you would like to reuse the flow cell, please follow the Flow Cell Wash Kit protocol and store the washed flow cell at 2-8°C.<br>☐ Alternatively, follow the returns procedure to flush out the flow cell ready to send back to Oxford Nanopore.	
IMPORTANT<br>☐ If you encounter issues or have questions about your sequencing experiment, please refer to the Troubleshooting Guide that can be found in the online version of this protocol.	
