# Supplementary material for: Automated environmental metagenomics using Oxford nanopore sequencing
Source: BMC Genomics. 2025 Sep 26;26:835. doi: 10.1186/s12864-025-11989-w (PMC12465296; doi:10.1186/s12864-025-11989-w)
Supplement: Supplementary file 3 — Additional file 3. Supplementary Figure 1. Analysis of medium-quality metagenome-assembled genomes. Supplementary Table 1. Table of read and assembly statistics for each sequenced sample. [file 12864_2025_11989_MOESM3_ESM.docx]

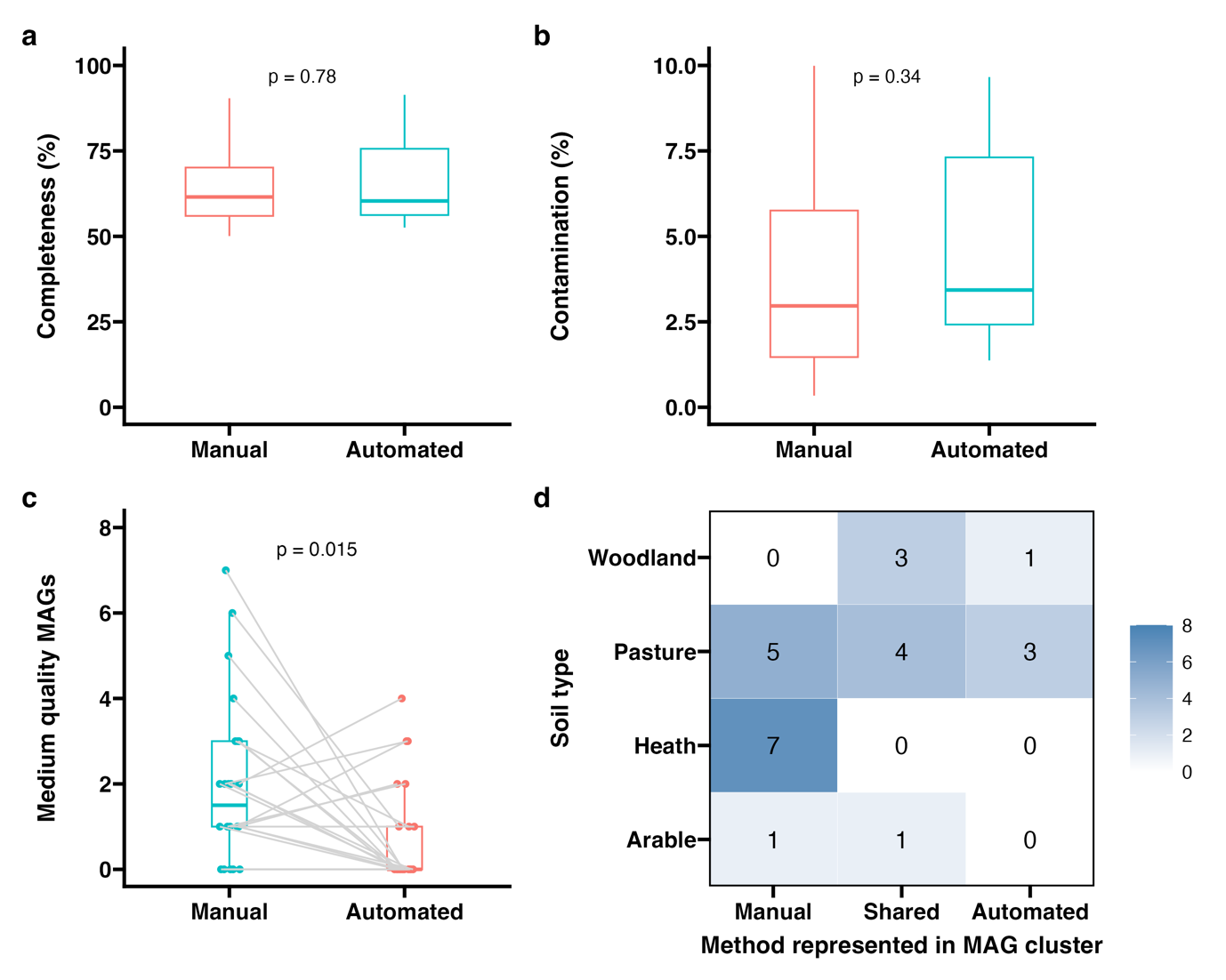


**Supplementary Figure 1. Analysis of medium-quality metagenome-assembled genomes.**

Boxplots comparing (a) completeness and (b) contamination across all medium- and high-quality metagenome-assembled genomes (MAGs) generated from manual or automated library preparation, with results of Wilcoxon signed-rank tests displayed. (c) Boxplot comparing the number of medium quality MAGs generated from each sample between parallel manual and automated libraries (indicated by grey lines), with the result of paired Wilcoxon signed-rank test displayed. (d) Heatmap displaying representation of samples from each library preparation method in dereplicated MAG clusters from each soil type, with clusters containing MAGs from both preparation methods.

**Supplementary Table 1. Sequencing raw read and assembly metrics**

Summary table of DNA sequencing raw read and assembly metrics for all automated and manual library preparations across four different soil habitats; Heath, Pasture, Woodland and Arable. Table displays total DNA sequence read count, average read length, maximum read length, sample N50, number of reads above Q20, average read Q score, percentage of reads classified, assembled contig N50 and number of medium quality MAGs generated.
